# Three-Dimensional Presentation of Tumor Histopathology: A Model Using Tongue Squamous Cell Carcinoma

**DOI:** 10.3390/diagnostics11010109

**Published:** 2021-01-12

**Authors:** Anne Koivuholma, Katri Aro, Antti Mäkitie, Mika Salmi, Tuomas Mirtti, Jaana Hagström, Timo Atula

**Affiliations:** 1 Department of Otorhinolaryngology-Head and Neck Surgery, Helsinki University Hospital and University of Helsinki, 00029 HUS Helsinki, Finland; katri.aro@hus.fi (K.A.); antti.makitie@hus.fi (A.M.); timo.atula@hus.fi (T.A.); 2 Department of Pathology, HUSLAB, Helsinki University Hospital and University of Helsinki, P.O. Box 21 Haartmaninkatu 3, 00290 Helsinki, Finland; tuomas.mirtti@hus.fi (T.M.); jaana.hagstrom@hus.fi (J.H.); 3 Department of Mechanical Engineering, Aalto University, P.O. Box 14100, Otakaari 4, 02150 Espoo, Finland; mika.salmi@aalto.fi; 4 Division of Ear, Nose, and Throat Diseases, Department of Clinical Sciences, Intervention, and Technology, Karolinska Institutet and Karolinska University Hospital, Stockholm, 171 64 Solna, Sweden; 5 Research Program in Systems Oncology, Faculty of Medicine, University of Helsinki, 00100 Helsinki, Finland; 6 Department of Oral Pathology and Radiology, University of Turku, 20520 Turku, Finland

**Keywords:** histopathology, three-dimensional reconstruction, computer-aided design, cancer, tongue

## Abstract

Medical imaging often presents objects in three-dimensional (3D) form to provide better visual understanding. In contrast, histopathology is typically presented as two-dimensional (2D). Our objective was to present the tumor dimensions in 3D by creating a 3D digital model of it and so demonstrate the location of the tumor and the histological slices within the surgical soft tissue resection specimen. We developed a novel method for modeling a tongue squamous cell carcinoma using commonly available instruments. We established our 3D-modeling method by recognizing and solving challenges that concern the selection of the direction of histological slices. Additional steps to standard handling included scanning the specimen prior to grossing and modeling the carcinoma, which required only a table scanner and modeling software. We present challenges and their solutions in modeling the resection specimen and its histological slices. We introduce a finished 3D model of a soft tissue resection specimen and the actual tumor as well as its histopathological grossing sites in 3D digital and printed form. Our novel method provides steps to create a digital model of soft tissue resection specimen and the tumor within. To our knowledge, this is the first attempt to present histopathological margins of a tongue tumor in 3D form, whereas previously, only 2D has been available. The creation of the 3D model does not call for predetermined grossing directions for the pathologist. In addition, it provides a crucial initiative to enhance oncological management. The method allows a better visual understanding of tumor margins, topography, and orientation. It thus provides a tool for an improved postoperative assessment and aids, for example, in the discussion of the need for additional surgery and adjuvant therapy.

## 1. Introduction

Three-dimensional (3D) presentation of various objects is common in engineering sciences and in medical imaging to enhance visual understanding of the object. In medical practice, however, histopathological samples are presented in two-dimensional (2D) slices. A few experiments have addressed this issue. These include two different methods to create a 3D representation of the tumor by creating a model, either from a series of 2D histological slides from the resection specimen or from preoperative 2D medical images. The former method has been used in modeling colorectal carcinoma and liver [1,2], radical prostatectomy [3,4], and bladder reconstruction [5], and the latter method for modeling lungs [6], breast cancer [7], pancreatic ductal adenocarcinoma [8], rectal cancer [9], and ghost cell odontogenic carcinoma [10]. Both methods have been used in modeling radical prostatectomy [11]. The methods thus far presented in the literature are not directly applicable to clinical practice since they require highly advanced modeling to create the 3D model. Furthermore, they do not allow the pathologist to select the slicing direction of the resection specimen freely because the 3D model is constructed from adjacent slices that require strictly predetermined slicing of the entire resection specimen. Typically, the pathologist slices the resection specimen more freely, generally using grossing directions perpendicular to each other and also targeted to critical areas or to a site of specific interest.

To our knowledge, none of the previous 3D models have included a soft tissue resection specimen combined with histology. Soft tissue specimens, such as a tongue tumor specimen, are asymmetric and prone to transform in shape after surgery, which makes them challenging to model. Our aim was to develop a method that is suitable for clinical practice with commonly available instruments, allows free selection of slicing direction, and is appropriate for soft tissue resection specimens. We describe various challenges encountered while developing the proposed method and suggest solutions for each technical phase. In our method, no additional slides are needed for recreating the 3D resection block and tumor, allowing the pathologist to choose grossing directions freely. The method yields a 3D visual representation of surgical margins and their directions. The method provides a tool for improving postoperative assessment of the need for additional surgery and adjuvant therapy.

## 2. Materials and Methods

**Figure 1:**
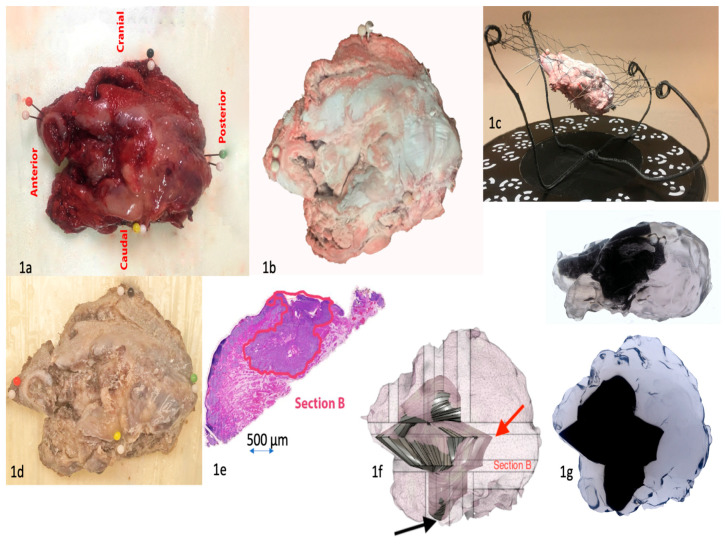
(**1a**) Resection specimen of the tongue with orientation. Needle colors indicate respective orientations, white needles are markers for scanning. (**1b**) Resection specimen in the scanning rack, top view. The specimen has been treated with white optic spray. (**1c**) Resection specimen in the scanning rack, side view. (**1d**) Resection specimen after formalin fixation and tissue coloring. (**1e**) Digitalized tumor outline (red) drawn by the pathologist. (**1f**) Digitalized resection specimen and modeled tumor. The closest margin in the resection specimen was 0.1 mm towards the bottom of the specimen (black arrow). This site was towards the neck, which was removed separately for technical reasons. The 3D model better demonstrates this site to the clinician. The largest margin was posteriorly 20 mm (red arrow). (**1g**) 3D printed object of the resection specimen and modeled tumor. The tumor has an angular appearance because its surface has been approximated as a plane between adjacent and perpendicular tumor outlines. If the number of histological slices is increased, hence increasing also the number of tumor outlines, the tumor surface will be smoother.

All study experiments were carried out at the Department of Pathology, HUSLAB, Helsinki University Hospital, and the University of Helsinki, Helsinki, Finland. To develop a practical method for creating the final 3D model, we first identified requirements for the modeling instruments and test materials. These included a scanner with a scanning platform, software compatible with the scanner, a test object, and the final object specimen. The most important requirement for the test object tissue material was that it has a texture similar to tongue tissue. Chicken fillet was chosen for the test tissue material, as it has texture, consistency, and reflectiveness close to human muscle tissue. A fictional tumor outline was modeled inside the chicken fillet digitally. For the final object, we chose a tongue squamous cell carcinoma (SCC) resection specimen (Figure 1a) because it exhibits well the challenges of soft tissue.

### 2.1. Software

We selected one piece of software to demonstrate applicability. The following requirements for the modeling software were assessed: It should be affordable, easy to use, and have the ability to handle triangulated surface models. For the data format, we selected .stl since most of the 3D scanners provide it, and it enables import and export of the 3D model between different pieces of software. Additional software for post-scanning processing is needed because commonly available table scanners do not provide software suitable for constructing the 3D model of the tumor.

Among various types of software, we selected six candidates for further evaluation: ImageJ (v2019, Wayne Rasband, open source) AutoCAD (v2019, Autodesk, Inc. Mill Valley, CA, USA), Recap Pro (v2019, Autodesk, Inc. Mill Valley, CA, USA), Tinkercad (v2019, Autodesk, Inc. Mill Valley, CA, USA), Rhinoceros (v2019, Robert McNeel & Associates, McNeel Europe, Barcelona, Spain) and Fusion 360 (v2019, Autodesk, Inc. Mill Valley, CA, USA). ImageJ and Tinkercad are free, whereas the annual costs for Fusion 360, AutoCAD, Recap Pro, and Rhinoceros vary from €350 to over €1000, depending on their properties. For this study, free trial periods were available to compare the pieces of software. Each piece of software can be downloaded directly online to a personal computer. ImageJ has been in use for reconstructing 3D models from histology slide stacks or magnetic resonance imaging (MRI) stacks. However, because ImageJ cannot import .stl files, it was rejected as a tool for creating the 3D model. ImageJ is widely used in handling very large digital pathology images. In our study, Image J was used to draw the digital outline of the tumor on the microscopic image, as it is in general use for this purpose at our institute. AutoCAD can import .stl files, and it is possible to model within the imported geometry, but the size of the imported file proved to be too large for AutoCAD to function properly. Tinkercad provided a more user-friendly interface than Fusion 360 or Rhinoceros, but the modeling tools were too basic for constructing the tumor efficiently. Recap Pro was tested for modifying the .stl file into a more workable form, but its design tools were less advanced than those of Fusion 360. Fusion 360 and Rhinoceros both fulfilled our criteria for 3D construction software. Fusion 360 was chosen because we found it to be more user-friendly (albeit with some less-advanced options) and more affordable than Rhinoceros.

Once Fusion 360 was selected, the next challenge was importing the scanned model into Fusion 360, as the .stl data file was very large. The scanner software creates a web of small triangles, i.e., mesh, that forms the surface geometry of the scanned object. In order for Fusion 360 to handle the scanned model, the web was reduced to 20,000 facets. This reduced the intricacy of the surface geometry. The web size level was assessed so that the geometry remained sufficiently fine for modeling the tumor. The tumor horizontal and vertical axes were placed to match the x, y, z-coordinate system in the Fusion 360 software.

### 2.2. Scanner and Scanning Procedure

Our requirements for the scanner were that it be inexpensive and portable. The scanned model should resemble the shape and size of the actual resection specimen as closely as possible, and the scanner should allow the model to be placed in any direction in a coordinate system. EinScan SP (Shining 3D) fulfilled our criteria and was selected for this study. EinScan SP costs around €2000 and has basic built-in software that makes it easy to import the scanned model in .stl form to modeling software. Einscan SP scanner has a turntable on which the object to be scanned is placed.

During scanning, the tissue surface showed reflections. If the surface is reflective, the scanning is impossible, as shiny areas are visualized as holes in the final model. At first, we tried to solve this problem by just padding the moisture off the surface, but this method was insufficient. Different kinds of powders, e.g., potato flour, were tested as a mattifying substance. However, flour proved to be quite messy, and it altered the surface geometry during scanning if it formed clusters. As a final solution, optic spray (Cerec Optispray, Sirona Dental Systems, LCC, Charlotte, NC, USA) was applied on the surface of the tissue specimen in order to prevent reflections. The spray was rinsed away with saline after scanning and before further processing of the tissue in order not to interfere with processing in the histopathology laboratory.

A major challenge with handling soft tissue is that it deforms easily. As a solution, we developed a metal rack with a flexible net in which the specimen is placed diagonally during scanning (Figure 1b,c). This procedure enabled us to place the specimen in an optimal position for scanning and to avoid flattening of the tissue. When placed on the net, the resection specimen “floats” in the air, allowing scanning from a wider surface area. Further, inclined placement of the specimen allows for more extensive scanning of the bottom and top than a horizontal position. The rack itself brought another challenge: How would it remain invisible in the final scan? By painting the rack matte black and by setting the scanner to optimally scan only light colors, the rack went undetected by the scanner.

One of the first challenges that we encountered in scanning the chicken fillet was how to indicate its precise orientation (anterior, posterior, ventral, dorsal) in the model. As a solution, the orientation of the specimen was marked with colored needles, similarly to our standard procedure with resection specimens (Figure 1a). However, white needles were required to be placed adjacent to the colored ones, as the colored needles remained undistinguishable in scanning.

**Figure 2:**
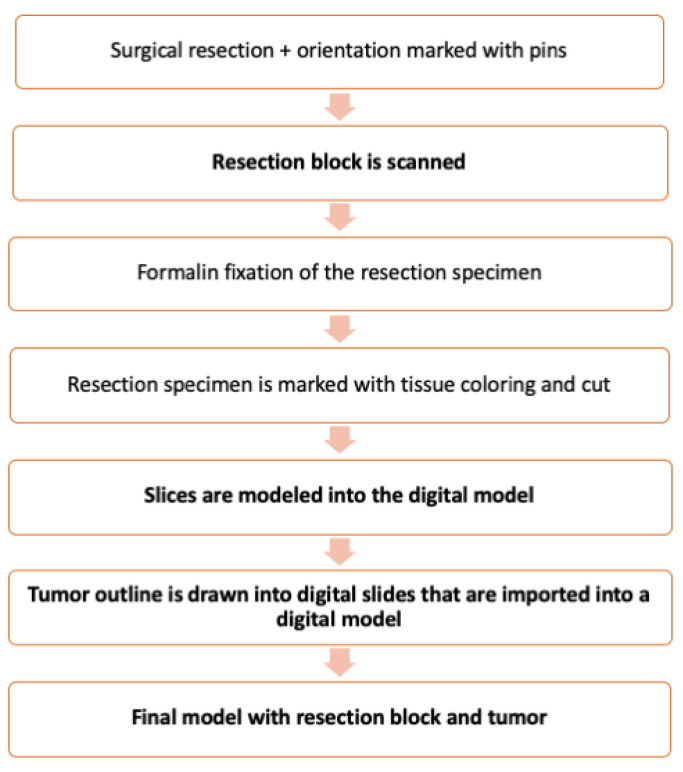
Flow chart of the method. The additions introduced by our method are in boldface.

Once it was proven that the scanner could create an accurate 3D model of the test tissue material (chicken fillet), the 3D model could be imported to Fusion 360, and the fictional tumor outline could be modeled inside the 3D test tissue image, the first patient case was tested. The method consists of two stages: First, modeling the resection specimen, and second, modeling the carcinoma within the resection specimen. The entire procedure is presented in Figure 2.

### 2.3. Experiments with Tongue Carcinoma Resection Specimen

We selected a typical tongue SCC (classified as T3 clinically and pathologically) for the first patient case. Resected tongue tissue is easily deformed, and this was likely to cause challenges in modeling.

With tongue tissue, after resection and marking with needles, the resection specimen was transported to the pathologist and scanned before it was fixed with formalin.

After formalin fixation (Figure 1d), the pathologist sliced the resection specimen in a normal manner, and the sliced blocks were embedded in paraffin. The challenge was to ensure that the histological slices could be aligned correctly in the 3D model. Therefore, before grossing the specimen to slices, all surfaces were marked with distinct tissue colors to indicate different directions. In addition, two thin lines with different colors were drawn, one on the dorsal surface and another perpendicular to it, allowing correct orientation and placement of the slices without distortion in the model. The pathologist cut the specimen into slices and marked the grossing sites onto the scanned resection specimen model in Fusion 360. The digital slices were cataloged in a standard fashion, e.g., the first slice in the anterior to posterior direction is named A, the second B, etc. The pathologist measured macroscopically the distance of the grossing sites from each border of the specimen as well as the thickness of the tissue slices.

We incorporated digital slices into the scanned model. First, the histopathological slides were scanned into a digital format. Second, the pathologist annotated the outline of the tumor onto the virtual microscopic image of each section using ImageJ software (Figure 1e). The bottom surface has a flat appearance (Figure 1e) due to the grossing of the resection specimen by the pathologist. Third, the tumor outline was matched with the respective digital slice in the digital specimen and aligned by using the color codes marked in Fusion 360. Fourth, once all the digital slices that included the digital tumor outline had been imported into the digital specimen, the tumor was modeled by combining the tumor outlines into a 3D body. Finally, the tumor was presented as a solid body surrounded by the resection specimen in a semitransparent form (Figure 1f). The final model can also be presented as a video.

### 2.4. D printing of the Resection Specimen Including the Tumor

The replica of the resection block, including the tumor, was 3D printed with a material jetting process using an Objet Connex 350 printer. The transparent material was RGD720, and the black material was VEROBLACKPLUS RGD875 with a layer thickness of 32 µm. The printed resection specimen and tumor are presented in Figure 1g.

The Research Ethics Board of the Hospital District of Helsinki and Uusimaa approved the study protocol (record number: HUS/1092/2018), and an institutional permit was granted.

## 3. Results

As a result, a final model of the resection specimen and tumor outline within the specimen is presented in semitransparent images (Figure 1f). An introductory video of the results can be seen at https://drive.google.com/file/d/19CdUuhDwPED5L3-e3fHKJsJPifLGbT6p/view?usp=sharing (accessed on 11 Jan 2021) (Supplementary Materials Video S1). Additionally, a 3D printed object of the resection block is presented in Figure 1g. The flow chart of the typical resection specimen processing with our additional steps is presented in Figure 2.

## 4. Discussion

In this study, we present a novel 3D modeling method that shows a soft tissue resection specimen, the actual tumor, the site of histological grossing, and the sites of histological slices within the specimen. Our method is suitable for soft tissue specimens, which are prone to distort during handling. Our method further allows the pathologist to choose the slicing direction of the resection specimen freely, which is crucial when various points of special interest of the specimen are required to assess, e.g., the surgical margins. Typically, the pathologist first cuts the resection block in half and determines the most crucial direction of the tumor growth. This then predetermines the direction of additional sections to be dissected. With our 3D model, the cut sections can be chosen freely and reconstructed in a three-dimensional space. After a few clarifications in the process, this method could be implemented into clinical use in the future as it optimizes visualization and understanding of tumor topography, location, and orientation and can thus be used to facilitate interdisciplinary discussion. This is a remarkable benefit and crucial since currently, only a written statement or a 2D image present the tumor margins. A 3D presentation is unambiguous and simple to interpret. Further, in our proposed method, it will only require a table scanner and modeling software to perform this.

Previous experimental methods for creating 3D histopathological models are based on either solely stacking subsequent histopathological slides or using medical imaging, or combining both [12]. When the model is constructed by digitally stacking histopathological slices, there is always a risk for a so-called banana effect [13]. This means that the histological slides are incorrectly stacked when reconstructed. If the original shape was a straight cylinder, the incorrectly stacked shape would be curved like a banana. When medical images, such as computer tomography (CT) scans, are utilized for the 3D reconstruction of the histopathological model, the problem is that the directions of the slices from imaging and histology need to be equal, and the specimen cannot be sliced in a normal manner since slicing of the specimen is predetermined and strict. Comparison between imaging scans and histopathological slides requires a number of histological slices, which means that the distance between the tissue grossing lines must be equivalent to the thickness of imaging slices [12]. Often, the slide thickness in an imaging scan is finer (1–3 mm) than the thickness of histological slides (3–5 mm). Thus, neither of these previous methods allows the pathologist to select the sectioning of the slices freely. In our method, we used a table scanner, which provides the surface geometry of the resection specimen, removing the risk of the banana effect. Our method allows the sample to be processed in a normal manner without the need for additional slices, and the pathologist can choose the grossing directions freely.

Presenting objects in a 3D form is a common tool in medical science, but with soft tissue, 3D tools are seldom applied. A major challenge with soft tissue reconstruction is how to retain the original shape of the resection specimen and the tumor. To minimize this problem of distortion, we developed a simple rack. The specimen rests somewhat undisturbed on the net of the rack. Still, the tissue is prone to withdraw from its original dimensions after resection. Future research is needed to resolve this issue, and we suggest a different kind of net and adding adhesive properties.

The reflection from the surface of the resection specimen posed a challenge during scanning. Our solution was eventually to use optic spray. The spray sticks to the surface of the tissue, rendering it matte, but not interfering with the surface geometry. We discovered that by spraying the colored needles, they also become visible after scanning. Thus, white needles can be used in future models to serve as a reference point. The optic spray did not interfere markedly with the tumor surface. As scanners are rapidly evolving, a technically more enhanced scanner might be able to take into account the reflectiveness and mobility of the soft tissues. One challenge that we observed in scanning was that the uneven surface of the resection specimen and the angle of scanning left some blind spots in the .stl model because the scanner beam is unable to reach every spot. This left two to three holes, approximately 1–5 mm in diameter, in the scan. After scanning, the EinScan SP software created a “watertight” model in which the remaining holes from the scan were modeled as solid. The software models these holes as smooth, solid planes. This process caused some loss of information from the original data.

Tissue shrinkage caused by formalin fixation must be taken into account in tumor measurements. The resection specimens are treated with formalin before the pathologist slices the specimen. The shrinkage in oral SCC varies between 14% and 24%, depending on orientation [14]. In this study, the resected specimen was scanned before formalin fixation, but the tumor was measured in a normal manner from histopathological slides after fixation. This difference can be corrected by scaling with an appropriate formalin shrinkage factor, in a similar manner as normally used when the pathologist assesses surgical margins. In our 3D digital model, the measurements can be scaled up by this factor in Fusion 360.

A number of stages in the handling process of the resection specimen during scanning, slicing, and modeling can induce inaccuracies in the finished model (Supplementary Materials Table S1). The pathologist cuts the resection specimen manually with a knife, thus the angle of the knife in relation to the specimen might not be completely straight. When the slices are imported into the model, it is assumed that the sliced cut with a knife is exactly straight. In addition, the slices are typically made in two directions, which are perpendicular to each other. As this grossing procedure is also done manually, the exact relation between the sections might not be perpendicular. Once the sections are modeled into the digital model, they are placed completely vertically or horizontally. This might cause distortion in the tumor model. This could be ameliorated by using some sort of a brace or scaffold when slicing the specimen. Furthermore, in order to place the tumor outline sections in proper orientation digitally, colored tissue markings in addition to the normal markings are needed. One streak was drawn in the horizontal direction of the resection specimen and another in a perpendicular direction. Tissue colors spread easily, and some colors are more difficult to detect under the microscope. An improved method for marking a narrow and sharp line would be helpful to avoid misalignment.

The carcinoma modeled in our study presented a sharp tumor border, and it was easy to mark explicitly inside the resection specimen model. If the tumor had an uneven border, the border area would be presented as tiny lumps without forming a uniform body. The geometry of the tumor was approximated as a linear line between adjacent tumor outlines marked on the virtual slices. In reality, the tumor might not follow a linear line, and the actual margin of the carcinoma might be somewhat different. However, this is an approximation that pathologists always use when analyzing tumor margins from a certain number of histopathological slices. In the future, artificial intelligence might be capable of detecting tumor outline automatically from virtual histopathological slices and generating the tumor automatically, greatly speeding up the process. Additionally, a tangible model of the tumor resection specimen and the tumor outline within could be constructed by using a 3D printer, as presented in our study.

Future steps for developing our method could include adding various histological elements or superimposing the construct with a 3D photomicrographic or radiological image. At the moment, our 3D model only includes the tumor outline and not the distinguishable histological elements of tissue, such as cells and vessels. This is due to the large size of the scanned histological slides that Fusion360 is unable to process. The evolvement of technology will most likely enable the inclusion of the histological representation into the 3D model. Our modeling method required only a few additional steps to the normal process. The use of the scanner allows rapid generation of the outline of the resection specimen after surgery, which enables fast generation of the 3D model. The scanning takes roughly 30 min, modeling of the grossing sites 5–15 min, and modeling of the tumor outline 15–60 min, depending on the case.

However, a familiarization period with the modeling software is necessary for the pathologist or laboratory technician. Like any new method in the experimental phase, it requires additional training and development. Still, we maintain that, with some simplification and automation, the method could be implemented into everyday use.

## 5. Conclusions

In this study, we presented a novel method for reconstructing a soft tissue tumor specimen and the carcinoma within it in a digital and 3D printed form. In addition to the normal process carried out by the pathologist, the extra tools required include a table scanner and software for the modeling. Unlike previously presented preliminary methods, our method allows free grossing directions, uses commonly available instruments, and does not require supplementary slices of the resection specimen. The method provides a better understanding of tumor margins, topography, and orientation. It serves as a tool for interdisciplinary discussion regarding postoperative assessment and adjuvant therapy. Our method requires familiarization with the software and scanner. Shrinkage of the soft tissue after resection is inevitable, should be taken into account, and evaluated in future studies. We conclude that this novel method could be implemented into clinical use with some additional development.

## Supplementary Materials

The following are available online at https://www.mdpi.com/2075-4418/11/1/109/s1, Table S1: Software selected for further evaluation in our study, Video S1: 3D histopathology demo.
