# Supplementary material for: Three-Dimensional Presentation of Tumor Histopathology: A Model Using Tongue Squamous Cell Carcinoma
Source: Diagnostics (Basel). 2021 Jan 12;11(1):109. doi: 10.3390/diagnostics11010109 (PMC7827352; doi:10.3390/diagnostics11010109)
Supplement: Supplementary file 1 [file diagnostics-11-00109-s001.zip › diagnostics-1037756-supple-for conversion/Supplementary Materials Table S1.pdf]

Table S1. Software selected for further evaluation in our study.

Software	Overview	Benefits	Challenges	Price Per Year*
Tinkercad	3D CAD design tool	Can import .stl ser-friendly interface	Basic modeling only	free
AutoCAD	3D CAD design tool	Can import .stl files	Unable to handle large .stl files	$1610 / 2227€
Recap PRO	3D models from scans	Can import .stl files	Meant for converting files into workable form	$350 / 355€
Fusion360	Advanced 3D design and model	Advanced modeling, .stl import	Requires familiarization	$495 / 503€
Rhinoceros	Advanced 3D design and model	Advanced modeling, .stl import	Requires familiarization	$995 / 995€
ImageJ	Meant for editing histopathological slides	Handles large microscopy images	Unable to import stl file	free

* pricing according to 2020.
